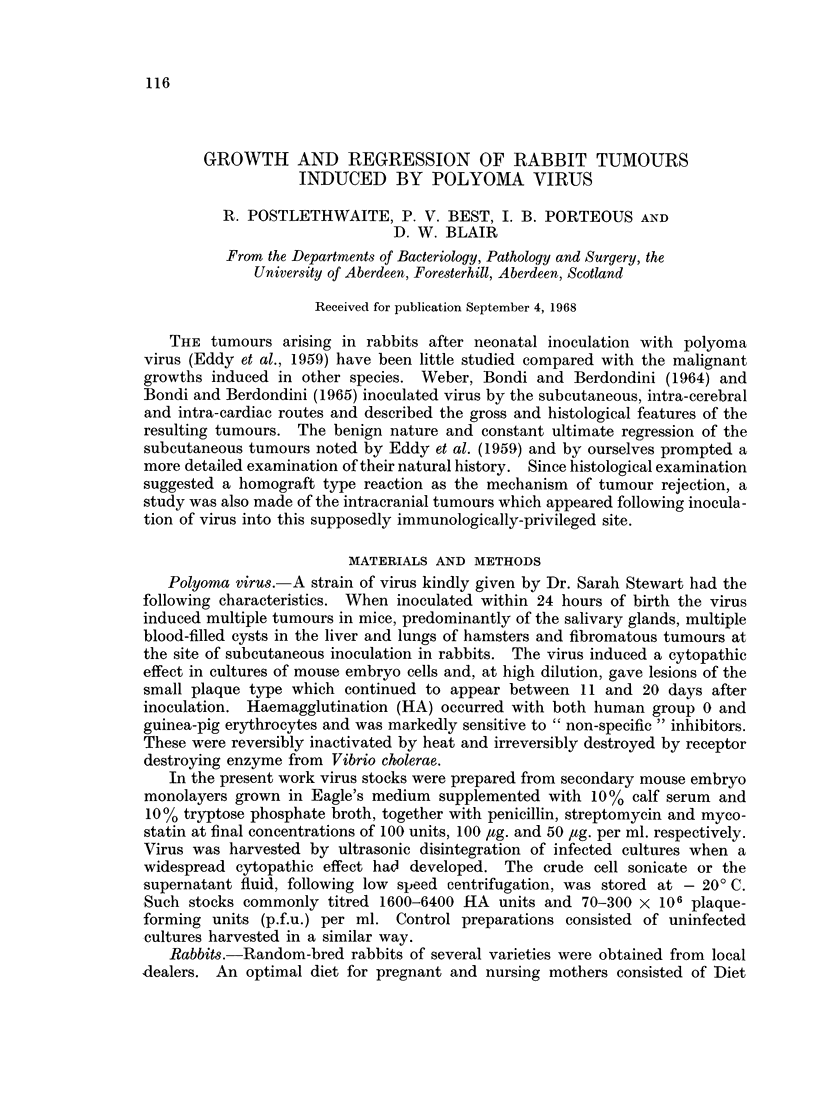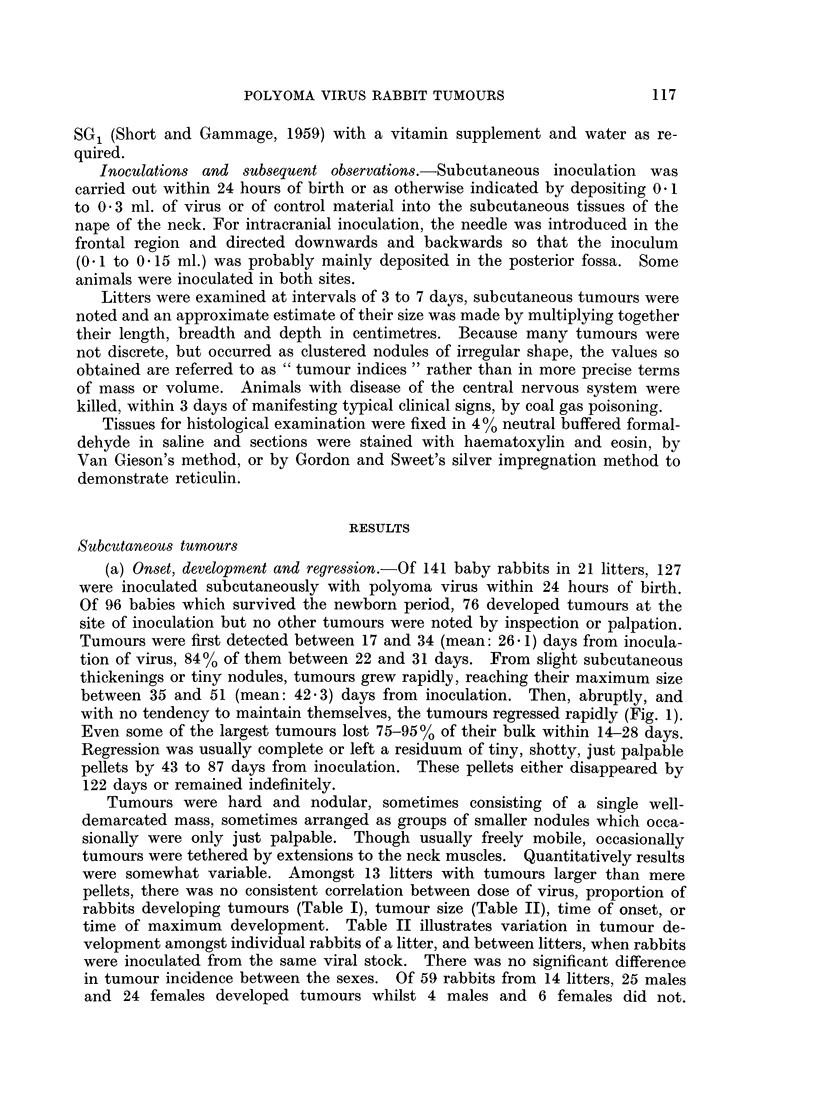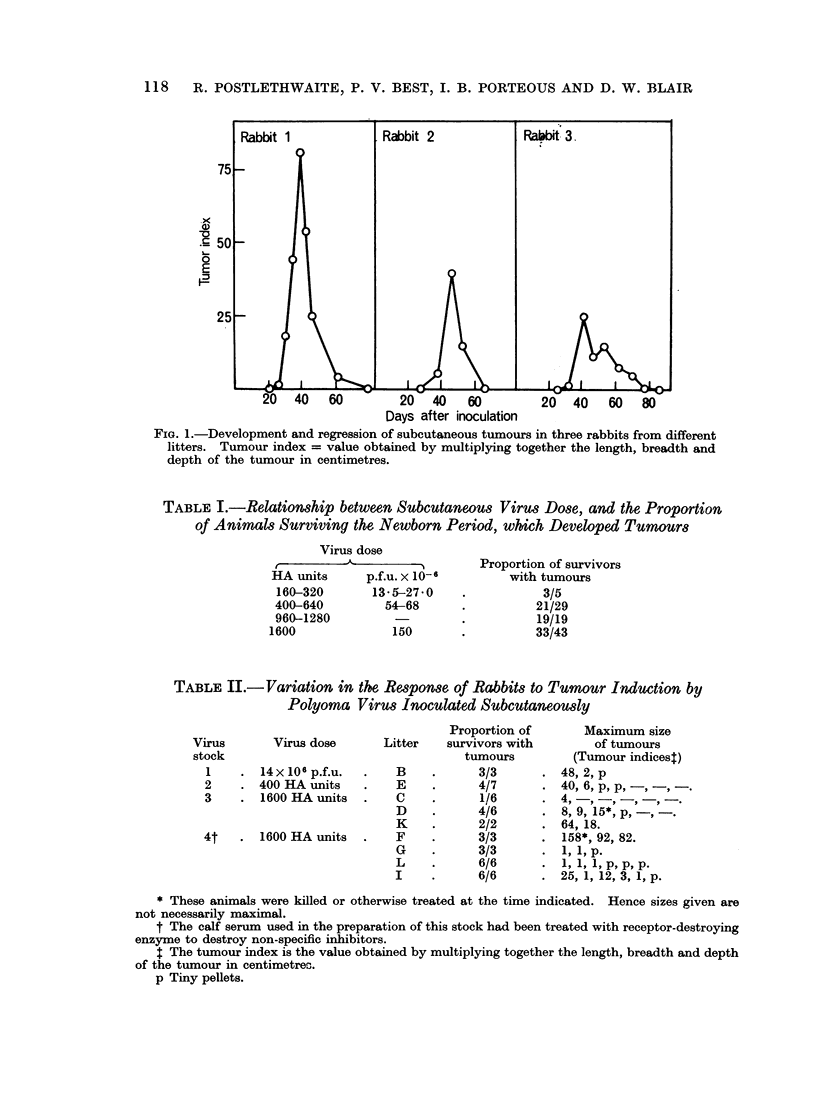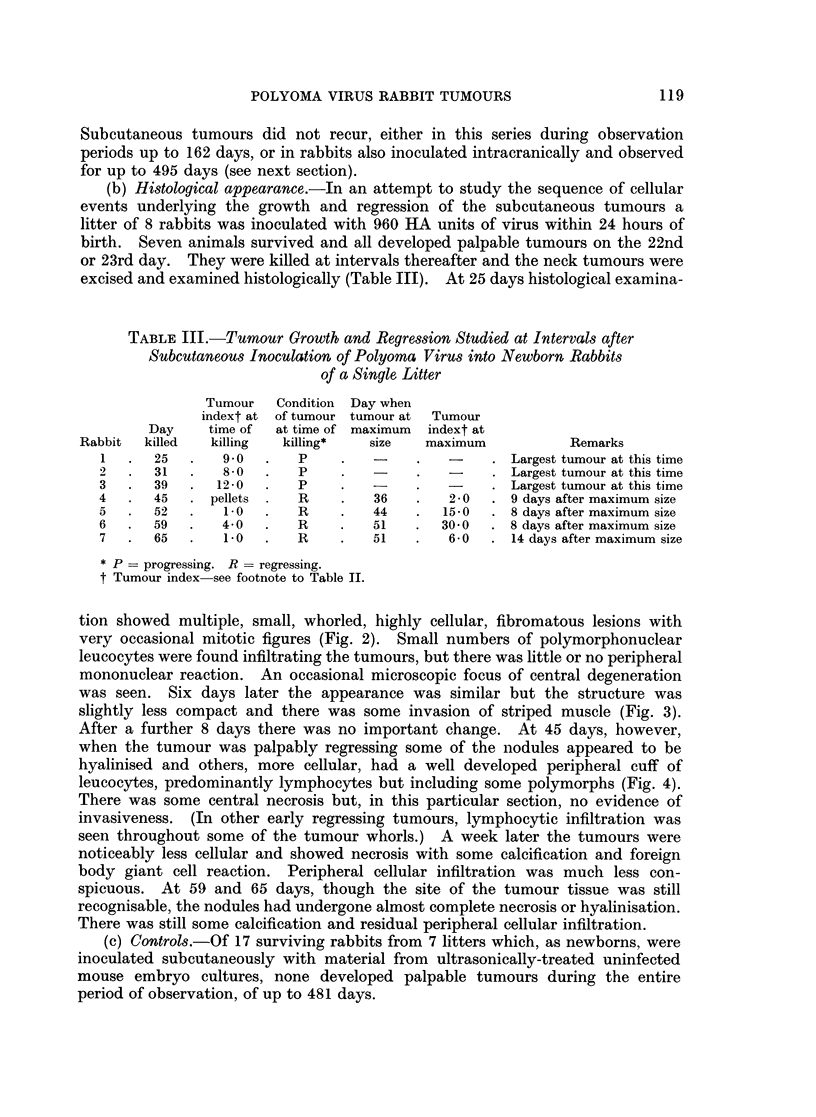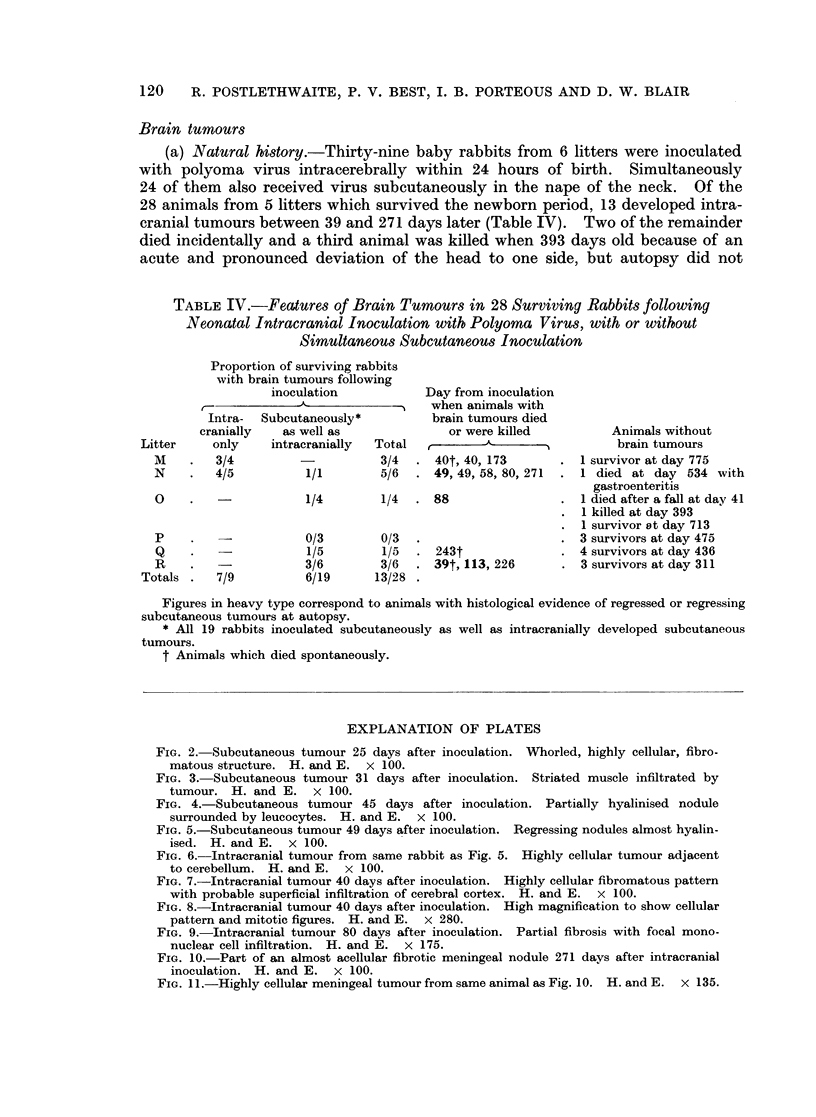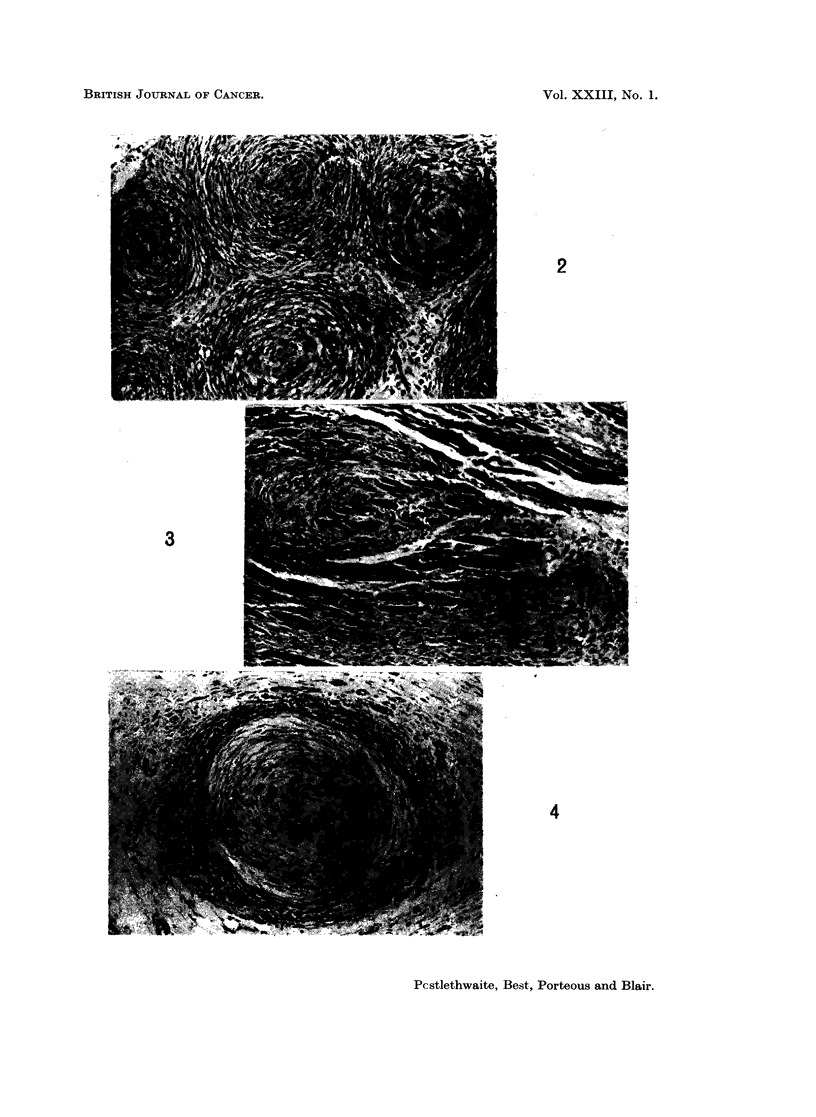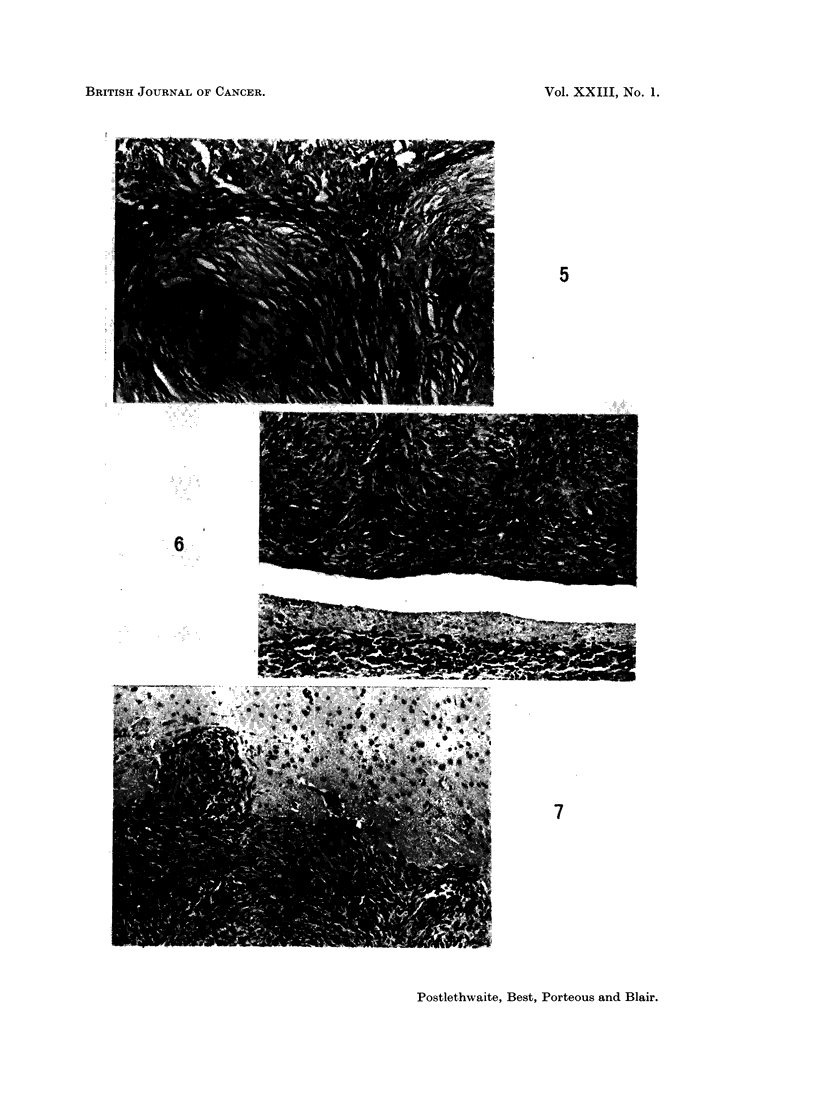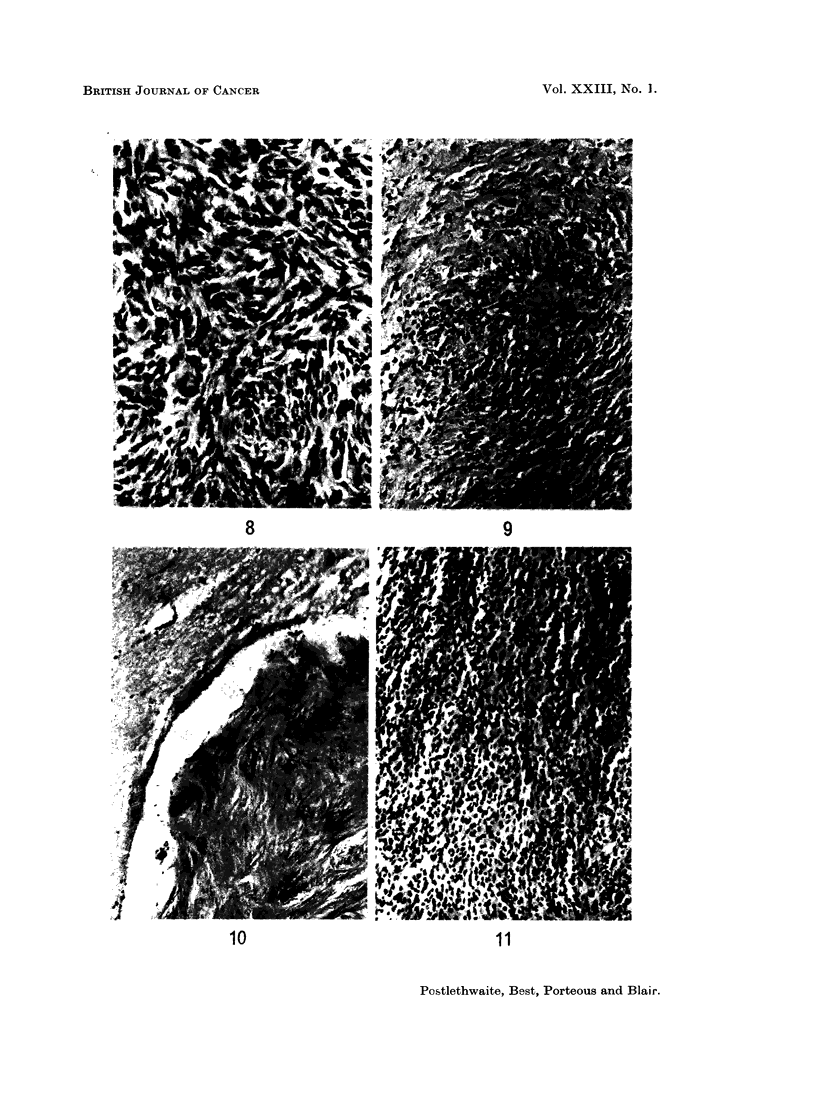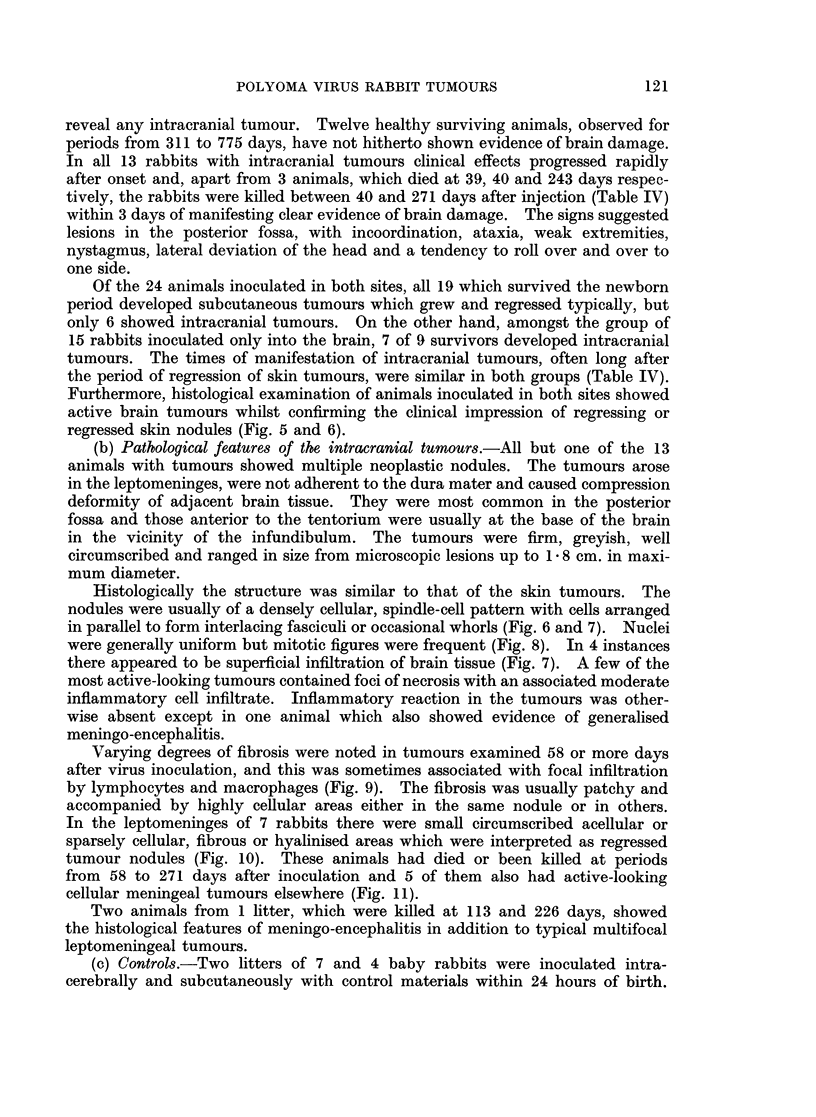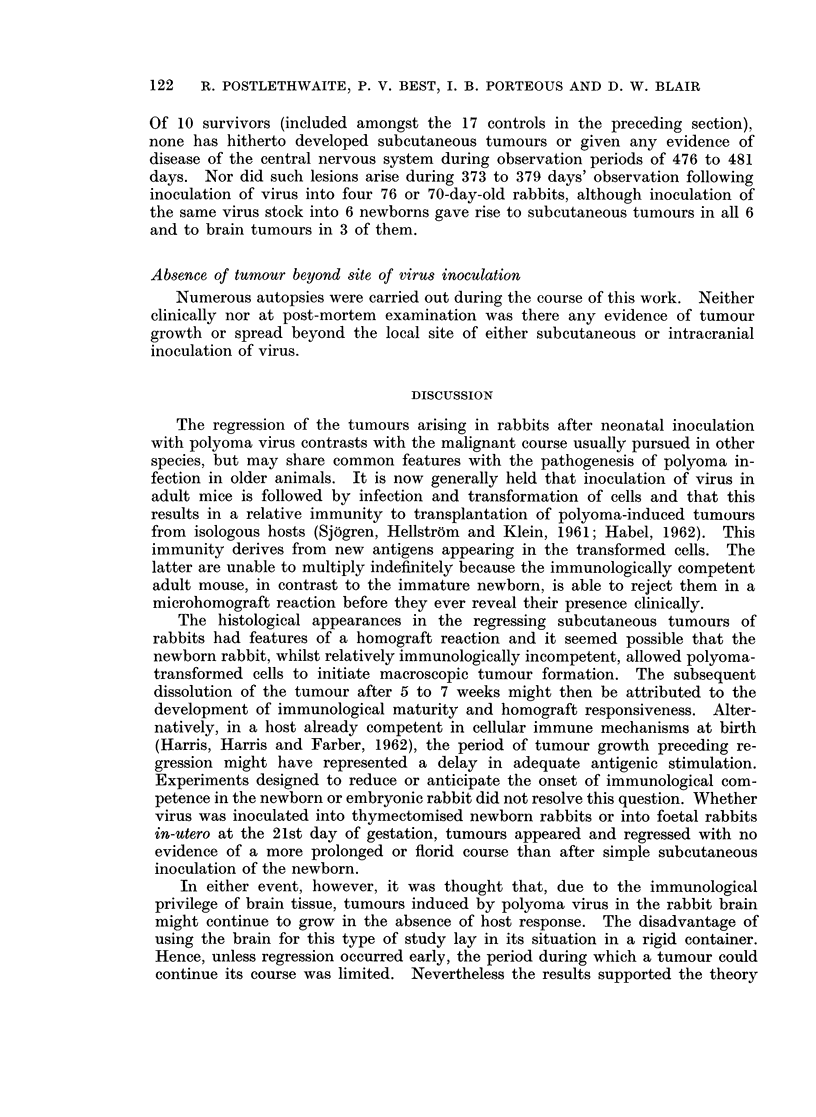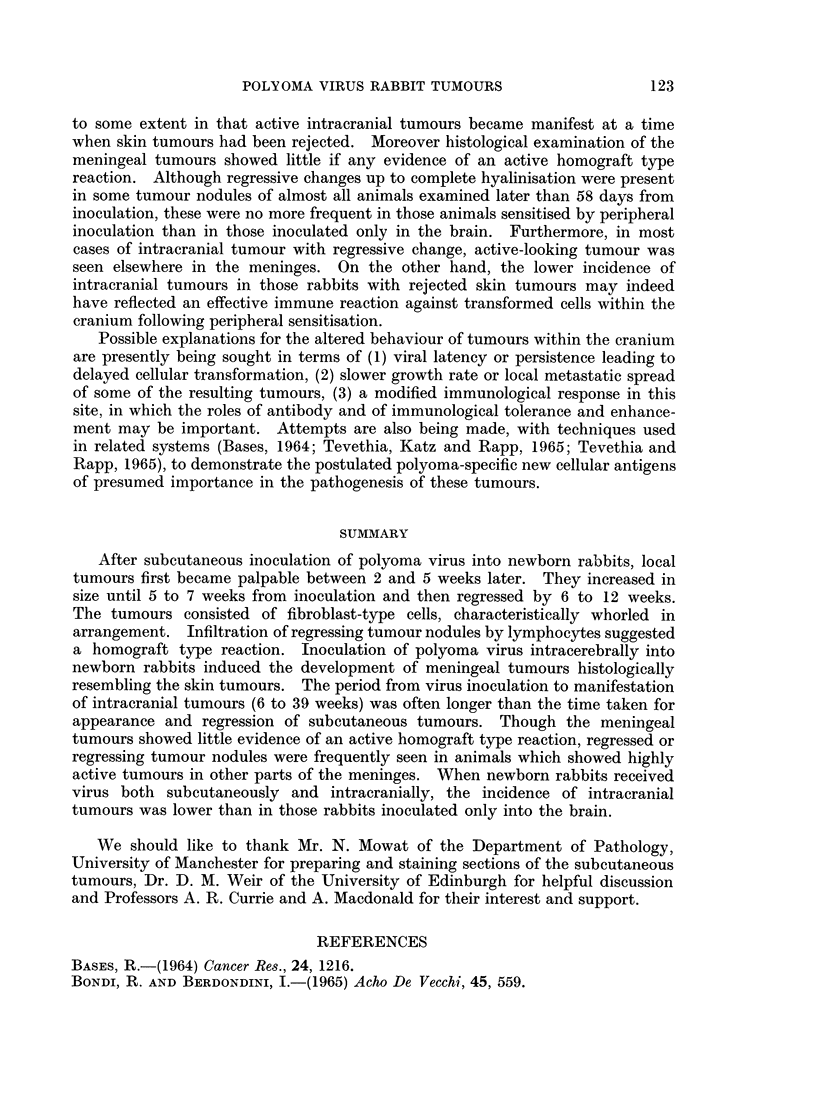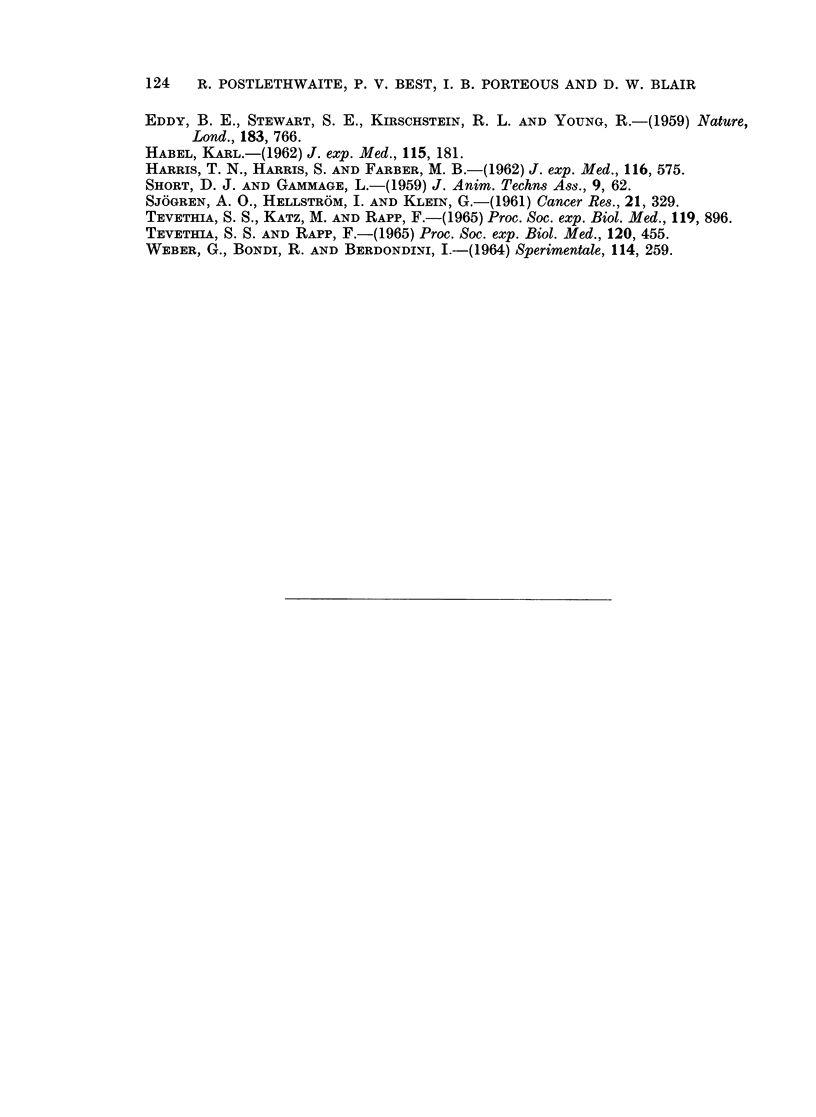# Growth and regression of rabbit tumours induced by polyoma virus.

**DOI:** 10.1038/bjc.1969.17

**Published:** 1969-03

**Authors:** R. Postlethwaite, P. V. Best, I. B. Porteous, D. W. Blair

## Abstract

**Images:**


### GROWTH AND REGRESSION OF RABBIT TUMOURS INDUCED BY POLYOMA VIRUS

R. POSTLETHWAITE, P. V. BEST, I. B. PORTEOUS AND D. W. BLAIR

*From the Departments of Bacteriology, Pathology and Surgery, the University of Aberdeen, Foresterhill, Aberdeen, Scotland*

Received for publication September 4, 1968

THE tumours arising in rabbits after neonatal inoculation with polyoma virus (Eddy *et al.*, 1959) have been little studied compared with the malignant growths induced in other species. Weber, Bondi and Berdondini (1964) and Bondi and Berdondini (1965) inoculated virus by the subcutaneous, intra-cerebral and intra-cardiac routes and described the gross and histological features of the resulting tumours. The benign nature and constant ultimate regression of the subcutaneous tumours noted by Eddy *et al.* (1959) and by ourselves prompted a more detailed examination of their natural history. Since histological examination suggested a homograft type reaction as the mechanism of tumour rejection, a study was also made of the intracranial tumours which appeared following inoculation of virus into this supposedly immunologically-privileged site.

### MATERIALS AND METHODS

*Polyoma virus.*—A strain of virus kindly given by Dr. Sarah Stewart had the following characteristics. When inoculated within 24 hours of birth the virus induced multiple tumours in mice, predominantly of the salivary glands, multiple blood-filled cysts in the liver and lungs of hamsters and fibromatous tumours at the site of subcutaneous inoculation in rabbits. The virus induced a cytopathic effect in cultures of mouse embryo cells and, at high dilution, gave lesions of the small plaque type which continued to appear between 11 and 20 days after inoculation. Haemagglutination (HA) occurred with both human group 0 and guinea-pig erythrocytes and was markedly sensitive to " non-specific " inhibitors. These were reversibly inactivated by heat and irreversibly destroyed by receptor destroying enzyme from *Vibrio cholerae.*

In the present work virus stocks were prepared from secondary mouse embryo monolayers grown in Eagle's medium supplemented with 10% calf serum and 10% tryptose phosphate broth, together with penicillin, streptomycin and mycostatin at final concentrations of 100 units, 100 $\mu$g. and 50 $\mu$g. per ml. respectively. Virus was harvested by ultrasonic disintegration of infected cultures when a widespread cytopathic effect had developed. The crude cell sonicate or the supernatant fluid, following low speed centrifugation, was stored at $-20°$ C. Such stocks commonly titred 1600–6400 HA units and 70–300 $\times 10^6$ plaque-forming units (p.f.u.) per ml. Control preparations consisted of uninfected cultures harvested in a similar way.

*Rabbits.*—Random-bred rabbits of several varieties were obtained from local dealers. An optimal diet for pregnant and nursing mothers consisted of Diet

$SG_1$ (Short and Gammage, 1959) with a vitamin supplement and water as required.

*Inoculations and subsequent observations.*—Subcutaneous inoculation was carried out within 24 hours of birth or as otherwise indicated by depositing 0·1 to 0·3 ml. of virus or of control material into the subcutaneous tissues of the nape of the neck. For intracranial inoculation, the needle was introduced in the frontal region and directed downwards and backwards so that the inoculum (0·1 to 0·15 ml.) was probably mainly deposited in the posterior fossa. Some animals were inoculated in both sites.

Litters were examined at intervals of 3 to 7 days, subcutaneous tumours were noted and an approximate estimate of their size was made by multiplying together their length, breadth and depth in centimetres. Because many tumours were not discrete, but occurred as clustered nodules of irregular shape, the values so obtained are referred to as " tumour indices " rather than in more precise terms of mass or volume. Animals with disease of the central nervous system were killed, within 3 days of manifesting typical clinical signs, by coal gas poisoning.

Tissues for histological examination were fixed in 4% neutral buffered formaldehyde in saline and sections were stained with haematoxylin and eosin, by Van Gieson's method, or by Gordon and Sweet's silver impregnation method to demonstrate reticulin.

### RESULTS

### *Subcutaneous tumours*

(a) *Onset, development and regression.*—Of 141 baby rabbits in 21 litters, 127 were inoculated subcutaneously with polyoma virus within 24 hours of birth. Of 96 babies which survived the newborn period, 76 developed tumours at the site of inoculation but no other tumours were noted by inspection or palpation. Tumours were first detected between 17 and 34 (mean: 26·1) days from inoculation of virus, 84% of them between 22 and 31 days. From slight subcutaneous thickenings or tiny nodules, tumours grew rapidly, reaching their maximum size between 35 and 51 (mean: 42·3) days from inoculation. Then, abruptly, and with no tendency to maintain themselves, the tumours regressed rapidly (Fig. 1). Even some of the largest tumours lost 75–95% of their bulk within 14–28 days. Regression was usually complete or left a residuum of tiny, shotty, just palpable pellets by 43 to 87 days from inoculation. These pellets either disappeared by 122 days or remained indefinitely.

Tumours were hard and nodular, sometimes consisting of a single well-demarcated mass, sometimes arranged as groups of smaller nodules which occasionally were only just palpable. Though usually freely mobile, occasionally tumours were tethered by extensions to the neck muscles. Quantitatively results were somewhat variable. Amongst 13 litters with tumours larger than mere pellets, there was no consistent correlation between dose of virus, proportion of rabbits developing tumours (Table I), tumour size (Table II), time of onset, or time of maximum development. Table II illustrates variation in tumour development amongst individual rabbits of a litter, and between litters, when rabbits were inoculated from the same viral stock. There was no significant difference in tumour incidence between the sexes. Of 59 rabbits from 14 litters, 25 males and 24 females developed tumours whilst 4 males and 6 females did not.

FIG. 1.—Development and regression of subcutaneous tumours in three rabbits from different litters. Tumour index = value obtained by multiplying together the length, breadth and depth of the tumour in centimetres.

TABLE I.—*Relationship between Subcutaneous Virus Dose, and the Proportion of Animals Surviving the Newborn Period, which Developed Tumours*

| Virus dose | | Proportion of survivors with tumours |
|---|---|---|
| HA units | p.f.u. × $10^{-6}$ | |
| 160–320 | 13·5–27·0 | 3/5 |
| 400–640 | 54–68 | 21/29 |
| 960–1280 | — | 19/19 |
| 1600 | 150 | 33/43 |

TABLE II.—*Variation in the Response of Rabbits to Tumour Induction by Polyoma Virus Inoculated Subcutaneously*

| Virus stock | Virus dose | Litter | Proportion of survivors with tumours | Maximum size of tumours (Tumour indices‡) |
|---|---|---|---|---|
| 1 | 14 × $10^6$ p.f.u. | B | 3/3 | 48, 2, p |
| 2 | 400 HA units | E | 4/7 | 40, 6, p, p, —, —, —. |
| 3 | 1600 HA units | C | 1/6 | 4, —, —, —, —, —. |
| | | D | 4/6 | 8, 9, 15*, p, —, —. |
| | | K | 2/2 | 64, 18. |
| 4† | 1600 HA units | F | 3/3 | 158*, 92, 82. |
| | | G | 3/3 | 1, 1, p. |
| | | L | 6/6 | 1, 1, 1, p, p, p. |
| | | I | 6/6 | 25, 1, 12, 3, 1, p. |

* These animals were killed or otherwise treated at the time indicated. Hence sizes given are not necessarily maximal.

† The calf serum used in the preparation of this stock had been treated with receptor-destroying enzyme to destroy non-specific inhibitors.

‡ The tumour index is the value obtained by multiplying together the length, breadth and depth of the tumour in centimetres.

p Tiny pellets.

Subcutaneous tumours did not recur, either in this series during observation periods up to 162 days, or in rabbits also inoculated intracranically and observed for up to 495 days (see next section).

(b) *Histological appearance.*—In an attempt to study the sequence of cellular events underlying the growth and regression of the subcutaneous tumours a litter of 8 rabbits was inoculated with 960 HA units of virus within 24 hours of birth. Seven animals survived and all developed palpable tumours on the 22nd or 23rd day. They were killed at intervals thereafter and the neck tumours were excised and examined histologically (Table III). At 25 days histological examina-

TABLE III.—*Tumour Growth and Regression Studied at Intervals after Subcutaneous Inoculation of Polyoma Virus into Newborn Rabbits of a Single Litter*

| Rabbit | Day killed | Tumour index† at time of killing | Condition of tumour at time of killing* | Day when tumour at maximum size | Tumour index† at maximum | Remarks |
|---|---|---|---|---|---|---|
| 1 | 25 | 9·0 | P | — | — | Largest tumour at this time |
| 2 | 31 | 8·0 | P | — | — | Largest tumour at this time |
| 3 | 39 | 12·0 | P | — | — | Largest tumour at this time |
| 4 | 45 | pellets | R | 36 | 2·0 | 9 days after maximum size |
| 5 | 52 | 1·0 | R | 44 | 15·0 | 8 days after maximum size |
| 6 | 59 | 4·0 | R | 51 | 30·0 | 8 days after maximum size |
| 7 | 65 | 1·0 | R | 51 | 6·0 | 14 days after maximum size |

* *P* = progressing. *R* = regressing.
† Tumour index—see footnote to Table II.

tion showed multiple, small, whorled, highly cellular, fibromatous lesions with very occasional mitotic figures (Fig. 2). Small numbers of polymorphonuclear leucocytes were found infiltrating the tumours, but there was little or no peripheral mononuclear reaction. An occasional microscopic focus of central degeneration was seen. Six days later the appearance was similar but the structure was slightly less compact and there was some invasion of striped muscle (Fig. 3). After a further 8 days there was no important change. At 45 days, however, when the tumour was palpably regressing some of the nodules appeared to be hyalinised and others, more cellular, had a well developed peripheral cuff of leucocytes, predominantly lymphocytes but including some polymorphs (Fig. 4). There was some central necrosis but, in this particular section, no evidence of invasiveness. (In other early regressing tumours, lymphocytic infiltration was seen throughout some of the tumour whorls.) A week later the tumours were noticeably less cellular and showed necrosis with some calcification and foreign body giant cell reaction. Peripheral cellular infiltration was much less conspicuous. At 59 and 65 days, though the site of the tumour tissue was still recognisable, the nodules had undergone almost complete necrosis or hyalinisation. There was still some calcification and residual peripheral cellular infiltration.

(c) *Controls.*—Of 17 surviving rabbits from 7 litters which, as newborns, were inoculated subcutaneously with material from ultrasonically-treated uninfected mouse embryo cultures, none developed palpable tumours during the entire period of observation, of up to 481 days.

*Brain tumours*

(a) *Natural history.*—Thirty-nine baby rabbits from 6 litters were inoculated with polyoma virus intracerebrally within 24 hours of birth. Simultaneously 24 of them also received virus subcutaneously in the nape of the neck. Of the 28 animals from 5 litters which survived the newborn period, 13 developed intracranial tumours between 39 and 271 days later (Table IV). Two of the remainder died incidentally and a third animal was killed when 393 days old because of an acute and pronounced deviation of the head to one side, but autopsy did not

TABLE IV.—*Features of Brain Tumours in 28 Surviving Rabbits following Neonatal Intracranial Inoculation with Polyoma Virus, with or without Simultaneous Subcutaneous Inoculation*

| Litter | Proportion of surviving rabbits with brain tumours following inoculation: Intracranially only | Subcutaneously* as well as intracranially | Total | Day from inoculation when animals with brain tumours died or were killed | Animals without brain tumours |
|---|---|---|---|---|---|
| M | 3/4 | — | 3/4 | 40†, 40, 173 | 1 survivor at day 775 |
| N | 4/5 | 1/1 | 5/6 | **49**, 49, 58, 80, 271 | 1 died at day 534 with gastroenteritis |
| O | — | 1/4 | 1/4 | **88** | 1 died after a fall at day 41 |
| | | | | | 1 killed at day 393 |
| | | | | | 1 survivor at day 713 |
| P | — | 0/3 | 0/3 | | 3 survivors at day 475 |
| Q | — | 1/5 | 1/5 | 243† | 4 survivors at day 436 |
| R | — | 3/6 | 3/6 | **39**†, **113**, 226 | 3 survivors at day 311 |
| Totals | 7/9 | 6/19 | 13/28 | | |

Figures in heavy type correspond to animals with histological evidence of regressed or regressing subcutaneous tumours at autopsy.

* All 19 rabbits inoculated subcutaneously as well as intracranially developed subcutaneous tumours.

† Animals which died spontaneously.

---

### EXPLANATION OF PLATES

FIG. 2.—Subcutaneous tumour 25 days after inoculation. Whorled, highly cellular, fibromatous structure. H. and E. × 100.

FIG. 3.—Subcutaneous tumour 31 days after inoculation. Striated muscle infiltrated by tumour. H. and E. × 100.

FIG. 4.—Subcutaneous tumour 45 days after inoculation. Partially hyalinised nodule surrounded by leucocytes. H. and E. × 100.

FIG. 5.—Subcutaneous tumour 49 days after inoculation. Regressing nodules almost hyalinised. H. and E. × 100.

FIG. 6.—Intracranial tumour from same rabbit as Fig. 5. Highly cellular tumour adjacent to cerebellum. H. and E. × 100.

FIG. 7.—Intracranial tumour 40 days after inoculation. Highly cellular fibromatous pattern with probable superficial infiltration of cerebral cortex. H. and E. × 100.

FIG. 8.—Intracranial tumour 40 days after inoculation. High magnification to show cellular pattern and mitotic figures. H. and E. × 280.

FIG. 9.—Intracranial tumour 80 days after inoculation. Partial fibrosis with focal mononuclear cell infiltration. H. and E. × 175.

FIG. 10.—Part of an almost acellular fibrotic meningeal nodule 271 days after intracranial inoculation. H. and E. × 100.

FIG. 11.—Highly cellular meningeal tumour from same animal as Fig. 10. H. and E. × 135.

2

3

4

Pcstlethwaite, Best, Porteous and Blair.

5

6

7

Postlethwaite, Best, Porteous and Blair.

8

9

10

11

Postlethwaite, Best, Porteous and Blair.

reveal any intracranial tumour. Twelve healthy surviving animals, observed for periods from 311 to 775 days, have not hitherto shown evidence of brain damage. In all 13 rabbits with intracranial tumours clinical effects progressed rapidly after onset and, apart from 3 animals, which died at 39, 40 and 243 days respectively, the rabbits were killed between 40 and 271 days after injection (Table IV) within 3 days of manifesting clear evidence of brain damage. The signs suggested lesions in the posterior fossa, with incoordination, ataxia, weak extremities, nystagmus, lateral deviation of the head and a tendency to roll over and over to one side.

Of the 24 animals inoculated in both sites, all 19 which survived the newborn period developed subcutaneous tumours which grew and regressed typically, but only 6 showed intracranial tumours. On the other hand, amongst the group of 15 rabbits inoculated only into the brain, 7 of 9 survivors developed intracranial tumours. The times of manifestation of intracranial tumours, often long after the period of regression of skin tumours, were similar in both groups (Table IV). Furthermore, histological examination of animals inoculated in both sites showed active brain tumours whilst confirming the clinical impression of regressing or regressed skin nodules (Fig. 5 and 6).

(b) *Pathological features of the intracranial tumours*.—All but one of the 13 animals with tumours showed multiple neoplastic nodules. The tumours arose in the leptomeninges, were not adherent to the dura mater and caused compression deformity of adjacent brain tissue. They were most common in the posterior fossa and those anterior to the tentorium were usually at the base of the brain in the vicinity of the infundibulum. The tumours were firm, greyish, well circumscribed and ranged in size from microscopic lesions up to 1·8 cm. in maximum diameter.

Histologically the structure was similar to that of the skin tumours. The nodules were usually of a densely cellular, spindle-cell pattern with cells arranged in parallel to form interlacing fasciculi or occasional whorls (Fig. 6 and 7). Nuclei were generally uniform but mitotic figures were frequent (Fig. 8). In 4 instances there appeared to be superficial infiltration of brain tissue (Fig. 7). A few of the most active-looking tumours contained foci of necrosis with an associated moderate inflammatory cell infiltrate. Inflammatory reaction in the tumours was otherwise absent except in one animal which also showed evidence of generalised meningo-encephalitis.

Varying degrees of fibrosis were noted in tumours examined 58 or more days after virus inoculation, and this was sometimes associated with focal infiltration by lymphocytes and macrophages (Fig. 9). The fibrosis was usually patchy and accompanied by highly cellular areas either in the same nodule or in others. In the leptomeninges of 7 rabbits there were small circumscribed acellular or sparsely cellular, fibrous or hyalinised areas which were interpreted as regressed tumour nodules (Fig. 10). These animals had died or been killed at periods from 58 to 271 days after inoculation and 5 of them also had active-looking cellular meningeal tumours elsewhere (Fig. 11).

Two animals from 1 litter, which were killed at 113 and 226 days, showed the histological features of meningo-encephalitis in addition to typical multifocal leptomeningeal tumours.

(c) *Controls*.—Two litters of 7 and 4 baby rabbits were inoculated intracerebrally and subcutaneously with control materials within 24 hours of birth.

Of 10 survivors (included amongst the 17 controls in the preceding section), none has hitherto developed subcutaneous tumours or given any evidence of disease of the central nervous system during observation periods of 476 to 481 days. Nor did such lesions arise during 373 to 379 days' observation following inoculation of virus into four 76 or 70-day-old rabbits, although inoculation of the same virus stock into 6 newborns gave rise to subcutaneous tumours in all 6 and to brain tumours in 3 of them.

*Absence of tumour beyond site of virus inoculation*

Numerous autopsies were carried out during the course of this work. Neither clinically nor at post-mortem examination was there any evidence of tumour growth or spread beyond the local site of either subcutaneous or intracranial inoculation of virus.

### DISCUSSION

The regression of the tumours arising in rabbits after neonatal inoculation with polyoma virus contrasts with the malignant course usually pursued in other species, but may share common features with the pathogenesis of polyoma infection in older animals. It is now generally held that inoculation of virus in adult mice is followed by infection and transformation of cells and that this results in a relative immunity to transplantation of polyoma-induced tumours from isologous hosts (Sjögren, Hellström and Klein, 1961; Habel, 1962). This immunity derives from new antigens appearing in the transformed cells. The latter are unable to multiply indefinitely because the immunologically competent adult mouse, in contrast to the immature newborn, is able to reject them in a microhomograft reaction before they ever reveal their presence clinically.

The histological appearances in the regressing subcutaneous tumours of rabbits had features of a homograft reaction and it seemed possible that the newborn rabbit, whilst relatively immunologically incompetent, allowed polyoma-transformed cells to initiate macroscopic tumour formation. The subsequent dissolution of the tumour after 5 to 7 weeks might then be attributed to the development of immunological maturity and homograft responsiveness. Alternatively, in a host already competent in cellular immune mechanisms at birth (Harris, Harris and Farber, 1962), the period of tumour growth preceding regression might have represented a delay in adequate antigenic stimulation. Experiments designed to reduce or anticipate the onset of immunological competence in the newborn or embryonic rabbit did not resolve this question. Whether virus was inoculated into thymectomised newborn rabbits or into foetal rabbits *in-utero* at the 21st day of gestation, tumours appeared and regressed with no evidence of a more prolonged or florid course than after simple subcutaneous inoculation of the newborn.

In either event, however, it was thought that, due to the immunological privilege of brain tissue, tumours induced by polyoma virus in the rabbit brain might continue to grow in the absence of host response. The disadvantage of using the brain for this type of study lay in its situation in a rigid container. Hence, unless regression occurred early, the period during which a tumour could continue its course was limited. Nevertheless the results supported the theory to some extent in that active intracranial tumours became manifest at a time when skin tumours had been rejected. Moreover histological examination of the meningeal tumours showed little if any evidence of an active homograft type reaction. Although regressive changes up to complete hyalinisation were present in some tumour nodules of almost all animals examined later than 58 days from inoculation, these were no more frequent in those animals sensitised by peripheral inoculation than in those inoculated only in the brain. Furthermore, in most cases of intracranial tumour with regressive change, active-looking tumour was seen elsewhere in the meninges. On the other hand, the lower incidence of intracranial tumours in those rabbits with rejected skin tumours may indeed have reflected an effective immune reaction against transformed cells within the cranium following peripheral sensitisation.

Possible explanations for the altered behaviour of tumours within the cranium are presently being sought in terms of (1) viral latency or persistence leading to delayed cellular transformation, (2) slower growth rate or local metastatic spread of some of the resulting tumours, (3) a modified immunological response in this site, in which the roles of antibody and of immunological tolerance and enhancement may be important. Attempts are also being made, with techniques used in related systems (Bases, 1964; Tevethia, Katz and Rapp, 1965; Tevethia and Rapp, 1965), to demonstrate the postulated polyoma-specific new cellular antigens of presumed importance in the pathogenesis of these tumours.

### SUMMARY

After subcutaneous inoculation of polyoma virus into newborn rabbits, local tumours first became palpable between 2 and 5 weeks later. They increased in size until 5 to 7 weeks from inoculation and then regressed by 6 to 12 weeks. The tumours consisted of fibroblast-type cells, characteristically whorled in arrangement. Infiltration of regressing tumour nodules by lymphocytes suggested a homograft type reaction. Inoculation of polyoma virus intracerebrally into newborn rabbits induced the development of meningeal tumours histologically resembling the skin tumours. The period from virus inoculation to manifestation of intracranial tumours (6 to 39 weeks) was often longer than the time taken for appearance and regression of subcutaneous tumours. Though the meningeal tumours showed little evidence of an active homograft type reaction, regressed or regressing tumour nodules were frequently seen in animals which showed highly active tumours in other parts of the meninges. When newborn rabbits received virus both subcutaneously and intracranially, the incidence of intracranial tumours was lower than in those rabbits inoculated only into the brain.

We should like to thank Mr. N. Mowat of the Department of Pathology, University of Manchester for preparing and staining sections of the subcutaneous tumours, Dr. D. M. Weir of the University of Edinburgh for helpful discussion and Professors A. R. Currie and A. Macdonald for their interest and support.

### REFERENCES

Bases, R.—(1964) *Cancer Res.*, **24**, 1216.
Bondi, R. and Berdondini, I.—(1965) *Acho De Vecchi*, **45**, 559.

EDDY, B. E., STEWART, S. E., KIRSCHSTEIN, R. L. AND YOUNG, R.—(1959) *Nature, Lond.*, **183**, 766.
HABEL, KARL.—(1962) *J. exp. Med.*, **115**, 181.
HARRIS, T. N., HARRIS, S. AND FARBER, M. B.—(1962) *J. exp. Med.*, **116**, 575.
SHORT, D. J. AND GAMMAGE, L.—(1959) *J. Anim. Techns Ass.*, **9**, 62.
SJÖGREN, A. O., HELLSTRÖM, I. AND KLEIN, G.—(1961) *Cancer Res.*, **21**, 329.
TEVETHIA, S. S., KATZ, M. AND RAPP, F.—(1965) *Proc. Soc. exp. Biol. Med.*, **119**, 896.
TEVETHIA, S. S. AND RAPP, F.—(1965) *Proc. Soc. exp. Biol. Med.*, **120**, 455.
WEBER, G., BONDI, R. AND BERDONDINI, I.—(1964) *Sperimentale*, **114**, 259.